# Supplementary figures and images for: Acetaldehyde breath test as a cancer risk marker in patients with esophageal and hypopharyngeal squamous cell carcinoma
Source: PLoS One. 2021 May 19;16(5):e0251457. doi: 10.1371/journal.pone.0251457 (PMC8133406; doi:10.1371/journal.pone.0251457)

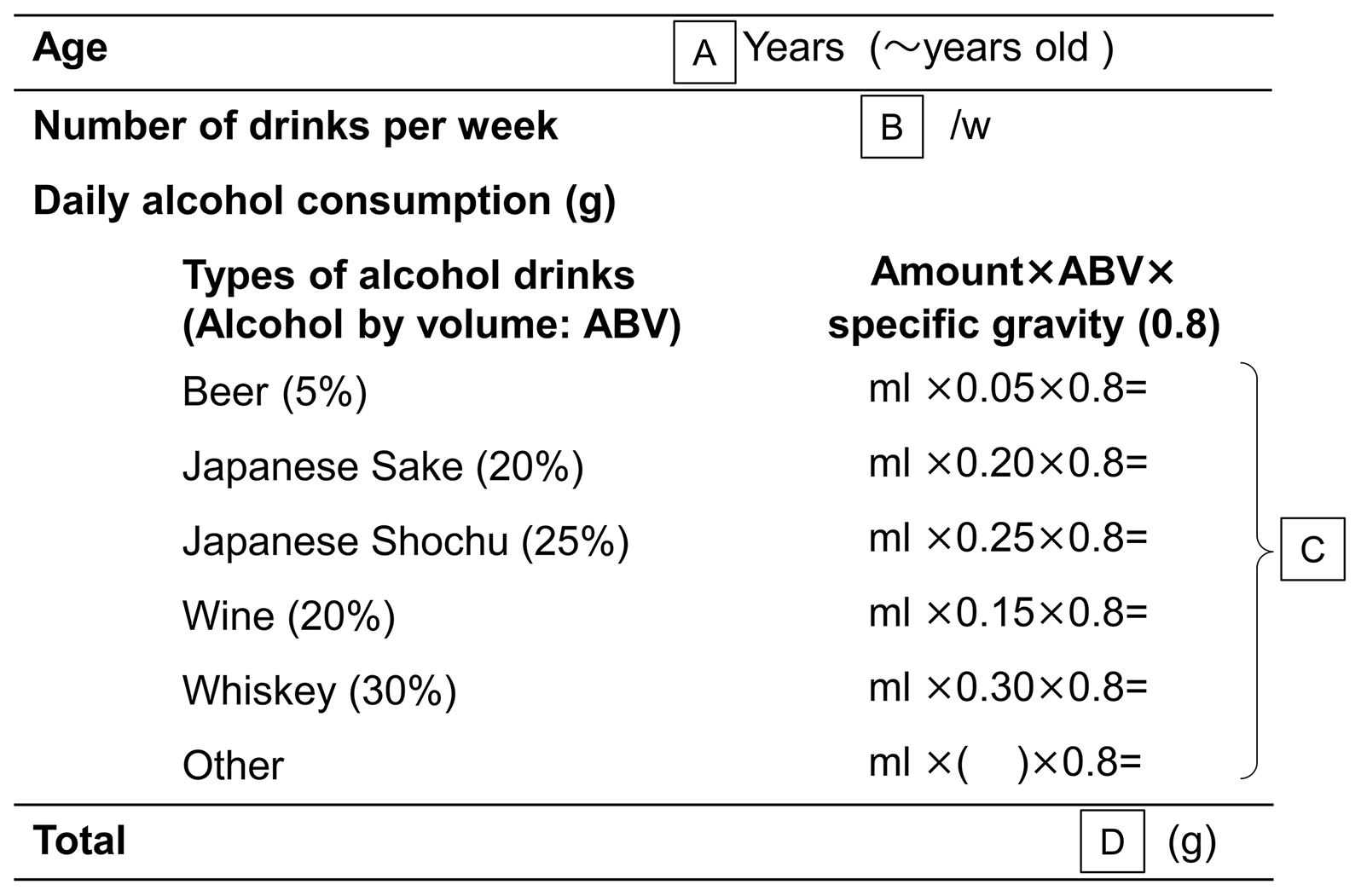

Supplement: S1 File — (TIF) [file pone.0251457.s001.tif]

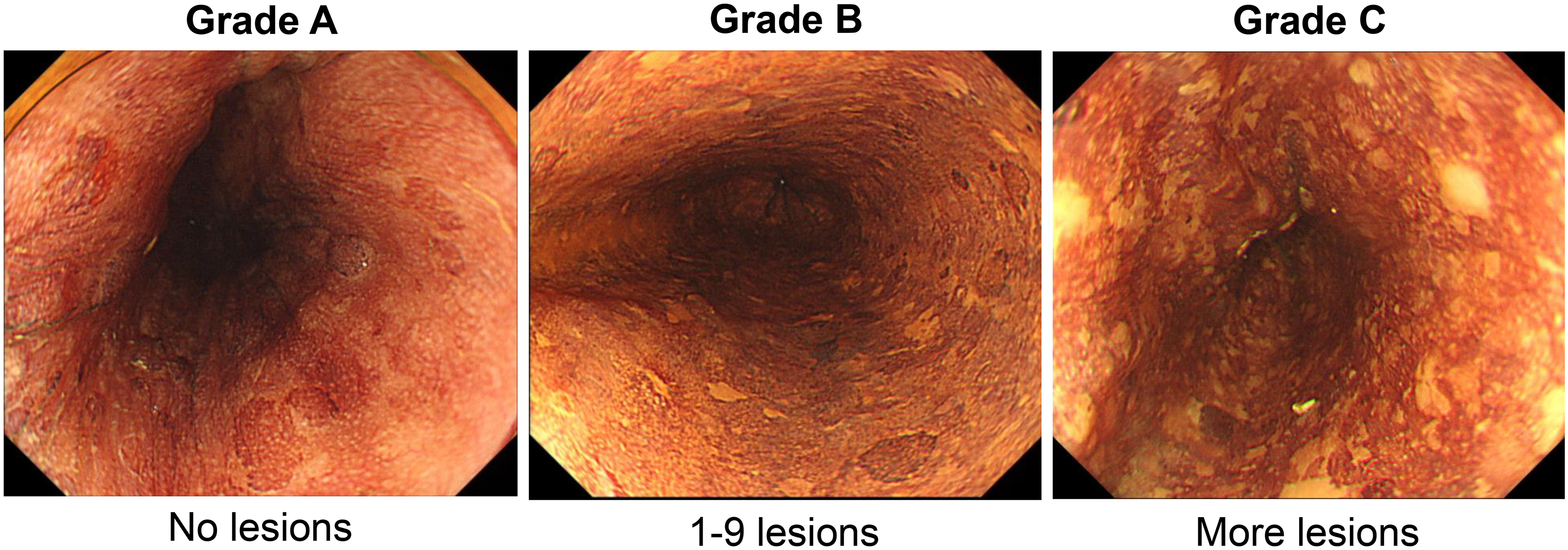

Supplement: S2 File — The number of LVLs per endoscopic view was counted, and the grading was divided into three categories. (TIF) [file pone.0251457.s002.tif]
