# Supplementary material for: Acetaldehyde breath test as a cancer risk marker in patients with esophageal and hypopharyngeal squamous cell carcinoma
Source: PLoS One. 2021 May 19;16(5):e0251457. doi: 10.1371/journal.pone.0251457 (PMC8133406; doi:10.1371/journal.pone.0251457)
Supplement: Attachment — Submitted filename: Response to Reviewer20210422.docx [file pone.0251457.s005.docx]

***PONE-D-21-01891***

***Acetaldehyde breath test as a cancer risk marker in patients with esophageal and hypopharyngeal squamous cell carcinoma***

***PLOS ONE***

***Review Comments to the Author***

***Reviewer #1: All comments were addressed. One suggestion would be to revise this introductory sentence for the relative activity of the ALDH2*1/*2 genotype- from the literature the activity is somewhere between 40-60% relative to wild type and <10% activity for a heterozygote relative to wild type ALDH2 seems to be a bit high.***

=> Thank you for your suggestion.I searched for papers on the activity of ALDH2*1/*2 and found two new papers reporting that ALDH2*1/*2 (heterozygous inactive form) is less than 20%. References 12 and 13 have been replaced.I was not able to find any papers that reported that ALDH2*1/*2 (heterozygous inactive form) is 40-60%.

***Reviewer #2: Minor comments:***

***Row 1/Title: Acetaldehyde breath test as a disease marker... Should be: ...as a cancer risk marker....***

=>Thank you for pointing this out. The title has been changed.

***Row 53: The latter prognosis word should be deleted***

=> Thank you for your suggestion. I deleted the latter prognosis word.

***Row 59: ...acetaldehyde dehydrogenase 2 (ALDH2) should be aldehyde dehydrogenase 2***

***Row 271: Reference number 34 is a review article and written in polish. Better to use the original article: Linderborg K et al. Food Chem Toxicol 2011,49:2103-06.***

=> Thank you for your suggestion. I corrected the points you raised.
